# Rapid Assessment of Avoidable Blindness in Western Rwanda: Blindness in a Postconflict Setting

**DOI:** 10.1371/journal.pmed.0040217

**Published:** 2007-07-03

**Authors:** Wanjiku Mathenge, John Nkurikiye, Hans Limburg, Hannah Kuper

**Affiliations:** 1 Rift Valley Provincial Hospital, Nakuru, Kenya; 2 London School of Hygiene and Tropical Medicine, London, United Kingdom; 3 King Faisal Hospital, Kigali, Rwanda; Hinchingbrooke Hospital, United Kingdom

## Abstract

**Background:**

The World Health Organization estimates that there were 37 million blind people in 2002 and that the prevalence of blindness was 9% among adults in Africa aged 50 years or older. Recent surveys indicate that this figure may be overestimated, while a survey from southern Sudan suggested that postconflict areas are particularly vulnerable to blindness. The aim of this study was to conduct a Rapid Assessment for Avoidable Blindness to estimate the magnitude and causes of visual impairment in people aged ≥ 50 y in the postconflict area of the Western Province of Rwanda, which includes one-quarter of the population of Rwanda.

**Methods and Findings:**

Clusters of 50 people aged ≥ 50 y were selected through probability proportionate to size sampling. Households within clusters were selected through compact segment sampling. Visual acuity (VA) was measured with a tumbling “E” chart, and those with VA below 6/18 in either eye were examined by an ophthalmologist. The teams examined 2,206 people (response rate 98.0%). The unadjusted prevalence of bilateral blindness was 1.8% (95% confidence interval [CI] 1.2%–2.4%), 1.3% (0.8%–1.7%) for severe visual impairment, and 5.3% (4.2%–6.4%) for visual impairment. Most bilateral blindness (65%) was due to cataract. Overall, the vast majority of cases of blindness (80.0%), severe visual impairment (67.9%), and visual impairment (87.2%) were avoidable (i.e.. due to cataract, refractive error, aphakia, trachoma, or corneal scar). The cataract surgical coverage was moderate; 47% of people with bilateral cataract blindness (VA < 3/60) had undergone surgery. Of the 29 eyes that had undergone cataract surgery, nine (31%) had a best-corrected poor outcome (i.e., VA < 6/60). Extrapolating these estimates to Rwanda's Western Province, among the people aged 50 years or above 2,565 are expected to be blind, 1,824 to have severe visual impairment, and 8,055 to have visual impairment.

**Conclusions:**

The prevalence of blindness and visual impairment in this postconflict area in the Western Province of Rwanda was far lower than expected. Most of the cases of blindness and visual impairment remain avoidable, however, suggesting that the implementation of an effective eye care service could reduce the prevalence further.

## Abstract 2

A survey of 2,250 people aged 50 y or over in Rwanda, based on clusters of 50 people, found a much lower prevalence of blindness than expected.

## Editors' Summary

**Background.:**

VISION 2020, a global initiative that aims to eliminate avoidable blindness, has estimated that 75% of blindness worldwide is treatable or preventable. The WHO estimates that in Africa, around 9% of adults aged over 50 are blind. Some data suggest that people living in regions affected by violent conflict are more likely to be blind than those living in unaffected regions. Currently no data exist on the likely prevalence of blindness in Rwanda, a central African country that is rebuilding following the 1994 genocide and civil war. Parts of the country, such as the Western Province, currently have no eye care services at all, but the government is trying to plan what services are necessary for this part of the country.

**Why Was This Study Done?:**

These researchers wanted to collect data that would help them estimate the number of people suffering from avoidable blindness in Western Province, Rwanda, and to find out the main causes of blindness in this region. The approach they adopted is known as the Rapid Assessment of Avoidable Blindness (RAAB).

**What Did the Researchers Do and Find?:**

This research project used survey methods based on the 2002 Rwandan national census. The researchers used the census to produce a list of settlements in Western Province, together with the number of individuals living in each settlement. Settlements were randomly picked from the list using a technique that was more likely to pick out bigger settlements than smaller ones. Each settlement was then divided into “cells,” with each cell containing around 500–700 people. One cell was randomly chosen from each settlement. Then, the researchers visited households within the cells, making sure that they visited 50 people aged over 50 y within each cell. They followed a standard procedure for collecting information from each person included in the survey. Each individual was examined by a nurse to measure their clearness of sight (“visual acuity”), using a Snellen chart (a chart with several rows of letters, where the size of the letters gets smaller as you go down the rows). The people being surveyed were examined by an ophthalmologist and the main cause of blindness was recorded, as well as general information on age, sex, details of any cataract operations, and why a cataract operation had not been done if one was needed.

Around 2 million people live in Western Province. The researchers included 2,250 people in the survey, for whom detailed examinations were done for 2,206 survey participants. Overall, 1.8% of the individuals examined were blind in both eyes. The main causes of blindness in the individuals surveyed were avoidable, and included cataract (clouding of the lens), focusing problems, and scarring of the cornea. Although 65% of cases of blindness were caused by cataract, and the availability of cataract surgery for those who needed it was reasonable, the outcomes of surgery were judged to be poor.

**What Do These Findings Mean?:**

>The overall proportion of individuals in this survey who were found to be blind was quite low—1.8% instead of the expected prevalence of 9%. The researchers estimated that the overall proportion of blind people in all age groups in this region of Rwanda would be around 0.2%, and they calculated that 365 cataract surgeries would be needed in the region every year to meet international targets for correcting cataracts. It is not clear why the prevalence of blindness was lower than expected in this survey; one factor might be the low proportion of people in the 50 y age group in the Rwandan population. However, this survey suggests that most of the cases of blindness in this population are avoidable, and the data produced here are important in planning future eye care services within Rwanda.

*PLoS Medicine*, as a leading general medical journal, would not usually publish the results of a survey of blindness (or any other medical condition) in just one part of one country. The editors felt this one was of particular interest for several reasons. There has previously been very little information about blindness prevalence in Rwanda. The idea of Rapid Assessment of Avoidable Blindness (RAAB) is also fairly new. Furthermore, the results are a striking contrast with what was found in two studies that we recently published from the southern Sudan (see below for references), another part of Africa that has experienced devastating conflict. The Sudan studies found a very much higher prevalence of blindness. However, it must be noted that the fighting in the Sudan continued over a much longer period (several decades) and the Sudanese environment is different in many respects; for example, it is much drier (which raises the risk of blindness due to trachoma) and many people live in extremely remote locations.

**Additional Information.:**

Please access these Web sites via the online version of this summary at http://dx.doi.org/10.1371/journal.pmed.0040217

- World Health Organization Health Topics maintains a minisite on blindness that includes links to fact sheets, statistics, official publications, and other information
- Wikipedia has an entry on visual acuity (clearness of sight), including details of how acuity is measured (note: Wikipedia is an internet encyclopedia anyone can edit)
- The World Health Organization publishes detailed country health profiles, including one for Rwanda (click on the relevant country name to download a PDF fact sheet)
- VISION 2020 is a global initiative aiming to eliminate avoidable blindness by the year 2020. Its Web site provides information on the main causes of avoidable blindness
- Two papers recently published in *PLoS Medicine* about blindness in the war-afflicted southern Sudan dealt with overall blindness prevalence (Ngondi J, Ole-Sempele F, Onsarigo A, Matende I, Baba S, et al. [2006] Prevalence and causes of blindness and low vision in southern Sudan. PLoS Med 3: e477) and blindness due to trachoma (Ngondi J, Ole-Sempele F, Onsarigo A, Matende I, Baba S, et al. [2006] Blinding trachoma in postconflict southern Sudan. PLoS Med 3: e478)

## Introduction

In 2004 the World Health Organization published the results of a major initiative to collate the available data on the prevalence of blindness [1]. On the basis of data from surveys in 55 countries, the authors estimated that there were approximately 37 million blind people in the world. Recent surveys, however, have reported a lower than expected prevalence of blindness [2–4], which may reflect a real decline in blindness or may result from improvements in survey quality. In contrast, a recent survey carried out in a postconflict area in Southern Sudan reported a prevalence of blindness of 4.1%, more than four times as high as expected for Africa as a whole [5]. There were methodological problems with this survey [6], but there are few other surveys available for postconflict areas to allow comparison. These data on the burden of blindness are needed to allow adequate planning and monitoring of eye care services. This is particularly true for low-income countries, where accurate data are needed to inform allocation of scarce resources.

The Republic of Rwanda is an extremely poor country in Eastern Central Africa with a population of 8.6 million. In 1994 a devastating war in Rwanda, following years of tension between the majority Hutus and minority Tutsis, resulted in the killing of nearly a million people and the exodus of more than two million. Although the war ended later the same year and many people have returned, today Rwanda is still suffering the aftermath of the genocide and mass population migrations. Despite its turbulent history, Rwanda is demographically similar to other African countries, with a large proportion (42%) of its population under the age of 15 years and a high rate of natural increase (2.5% per year). The Western Province of Rwanda has a population of around 2 million people and makes up one-quarter of the population of Rwanda. It was particularly badly affected by the war as a large proportion of its population was killed, and it still suffers from unrest.

No survey of blindness has been conducted in Rwanda to date. Results from the 2002 national census suggest that 13,100 of the total population of 8.1 million were blind, giving a prevalence of blindness of 0.16% [7]. This is likely to be an underestimate, as the census enumerators may have defined blindness as the complete inability to see, rather than the standard definition of VA less than 3/60 in the better eye. Meanwhile, the WHO data suggest that 1% of all people in Rwanda are blind, corresponding to 86,000 people, and that 9% of people aged over 50 years are blind [1].

The availability of ophthalmic services is extremely limited in Rwanda. There are only ten ophthalmologists in Rwanda, five of whom are based in the capital Kigali. In 2005, 2,600 cataract surgeries were undertaken in Rwanda, to give a cataract surgical rate (i.e., operations per million population per year) of only 300, far lower than the target for Africa of approximately 2,000 [8]. Ophthalmic services are particularly scarce in the Western Province, where there is no ophthalmologist or full-time eye clinic and many people go to neighbouring Democratic Republic of the Congo (another war-torn area) to seek care. Eye care services are currently being planned for the Western Province, so prevalence estimates of the burden of disease are needed urgently.

The aim of this study was to conduct a Rapid Assessment of Avoidable Blindness (RAAB) [2,3], in people aged 50 years or above in the Western Province of Rwanda, to allow planning of eye care services for this population.

## Methods

### Sample Size Calculation

No population-based surveys of blindness have been conducted in Rwanda. WHO estimates that the expected prevalence of blindness in countries in Africa is 9% in people aged ≥ 50 y [1]. Conservatively estimating the prevalence of blindness in people aged ≥ 50 y to be 7%, with the worst acceptable result of 5.6% (i.e., precision of 20%), 95% confidence, design effect (DEFF) of 1.6, and a non-response rate of 10%, a sample size of 2,229 was required, which would require 45 clusters of 50 people aged ≥ 50 y.

### Sampling Frame

The clusters were selected through probability proportionate to size sampling, using updated data from the 2002 national census as the sampling frame [7]. A list was produced of the enumeration areas (or secteurs, usually corresponding to a defined settlement in the rural areas or urban suburb) and their respective population sizes of people aged ≥ 50 y, based on the population distribution from the census. A column was created with the cumulative population across the enumeration areas. The total population age ≥ 50 y was divided by the number of clusters required to derive the sampling interval. The first cluster was selected by multiplying the sampling interval with a random number between 0 and 1, the resulting number was traced in the cumulative population column, and the first cluster was taken from the corresponding enumeration area. The following clusters were identified by adding the sampling interval to the previous number.

The second stage, of selecting households within clusters, was undertaken through compact segment sampling [9]. Each cluster area (the enumeration area) was divided into segments called cells for administrative purposes. Each cell included approximately 500–700 people, of whom around 50 people were expected to be aged ≥ 50 y. The cells had well-defined boundaries, and the names of all inhabitants were known by the village head. One cell in the cluster area was chosen at random by drawing lots. Starting at the edge of the segment, all households in the cell were included in the sample sequentially, until 50 people aged ≥ 50 y were identified. Eligible participants were those aged ≥ 50 y who lived in the household for at least three months of the year. If the segment did not include 50 people aged ≥ 50 y, then another segment was chosen at random and sampling continued until the required cluster size of 50 was achieved**.**

The survey was carried out during four weeks from 29 May until 24 June 2006. In each cluster, the survey team visited households door-to-door, accompanied by a local village guide. The team conducted the visual examinations in the household. If an eligible person was absent, the survey team returned twice to the household on the same day to examine the individual before leaving the area. If after repeated visits the individual could not be examined, information about the visual status was collected from relatives or neighbours.

### Ophthalmic Examination

A standardised protocol was used for the RAAB [2,3]. A survey record was completed for each eligible person that included seven sections: general demographic information; vision and pinhole examination; lens examination; principal cause of visual impairment; history of visual impairment; why cataract operation had not been done; and details about any cataract operation.

VA was measured by a nurse with ophthalmic experience, with a Snellen tumbling “E” chart using optotype size 6/18 (20/60) on one side and size 6/60 (20/200) on the other side at a distance of 6 or 3 m. All measurements were taken in full daylight with available spectacle correction. If the VA was less than 6/18 in either eye, then pinhole vision was also measured. Categories of visual impairment were defined as follows. Blindness, VA < 3/60 in the better eye with available correction; severe visual impairment, VA ≥ 3/60 but < 6/60 in the better eye with available correction; and visual impairment, VA ≥ 6/60 but < 6/18 in the better eye with available correction.

All participants were examined by an ophthalmologist. The lens status was assessed by torch and by distant direct ophthalmoscopy in a shaded or dark environment without dilatation of the pupil. Lens status was graded as: “normal lens”, “obvious lens opacity present”, “lens absent (aphakia)”, or “IOL implantation”. If the lens could not be examined (e.g. corneal scarring present) then “no view of lens” was noted. The ophthalmologist examined all eyes of individuals with a presenting VA < 6/18 with a flashlight and a direct ophthalmoscope. The examination was made with pupil dilation if the cause of visual impairment was not refractive error, cataract, aphakia, or corneal scar. Since detailed ophthalmic examinations, including measurement of visual fields and intraocular pressure, were not possible in this door-to-door survey, diabetic retinopathy, glaucoma, age-related macular disease, and other types of posterior segment disease were categorised together as “posterior segment disease.” The principal cause of blindness or visual impairment was recorded, according to the WHO convention where the major cause is assigned to the primary disorder or, if there are two or more existing primary disorders contributing equally to the visual impairment, to the one that is easiest to treat [10]. People who had undergone cataract surgery were asked about the date and place of operation and their level of satisfaction with the surgery.

### Training

There were three teams, each consisting of one ophthalmologist and one nurse from an eye unit. The teams received one week of training by an experienced trainer. Interobserver agreement was measured through repeat examination of 40 patients by each of the three teams. Measurement of VA, examination of lenses, and assignment of cause of blindness were compared between the teams to ensure that their standards were acceptable (i.e., kappa ≥ 0.60).

### Statistical Analysis

A software programme developed for this survey (RAAB test version 4.00 developed in Visual FoxPro v. 7.0) was used for data entry and automatic standardised data analysis. The prevalence estimates took account of the DEFF when estimating the confidence intervals. Estimates of the number of cases with blindness, severe visual impairment, and visual impairment were obtained by extrapolating the age- and sex-specific prevalence estimates to the age and sex structure of the population of the Western Province. The cataract surgical coverage (CSC) of people is the proportion of people needing surgery that had undergone cataract surgery. This is calculated as the number of people who have had cataract surgery divided by the number of people who need cataract surgery. It is calculated by dividing the number of people with bilateral pseudophakia/aphakia plus those with unilateral pseudophakia/aphakia and visual impairment in other eye, by those who have undergone surgery (i.e., the same number above) plus the number of people with bilateral visual impairment from cataract. CSC was also calculated for eyes. Since the VA prior to surgery was not known, we assumed, in turn, that only patients with VA below a certain threshold (<3/60, <6/60, and <6/18, respectively), received surgery for their cataract.

### Ethical Approval

Ethical approval for this work was granted by the Ministry of Health, administrative heads of the Western Province, and The National Statistics Council. Informed verbal consent was obtained from the patients after explanation of the nature and possible consequences of the study. All people with operable cataract or other treatable conditions were referred for treatment.

## Results

The teams enumerated 2,250 people aged ≥ 50 y in the survey, of whom 2,206 (98.0%) underwent ophthalmic examination, 43 (1.9%) were not available, and one (0.04%) refused to be examined. The examined population was representative of the population of the province in terms of age and gender distribution (Table 1).

**Table 1:**
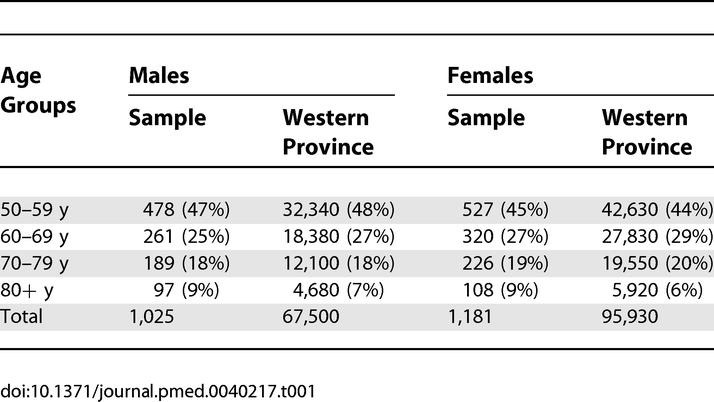
Age and Gender Composition of Population in Sample and in Survey Area

The unadjusted prevalence of blindness in the examined population aged ≥ 50 y was 1.8% (95% confidence interval [CI] 1.2%–2.4%; DEFF = 1.1), which was similar in men and women (Table 2). The prevalence of severe visual impairment was also low (1.3%, 95% CI 0.8%–1.7%; DEFF = 0.9), as was the prevalence of visual impairment (5.3%, 95% CI 4.2%–6.4%; DEFF = 1.4). Only 22 people had aphakia or pseudophakia in one or both eyes.

**Table 2:**
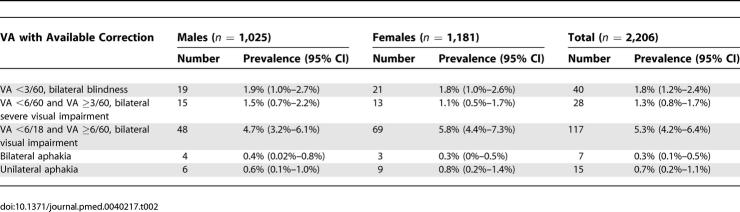
Distribution by Visual Acuity with Available Correction in the Better Eye in Adults Aged 50 Years and Older

The majority of cases of blindness (65.0%), severe visual impairment (60.7%), and visual impairment (54.7%) were attributable to cataracts (Table 3). Posterior segment disease was an important cause of blindness (20.0%) and severe visual impairment (32.1%), while refractive error caused almost one-third of cases of visual impairment (29.9%). Overall, the vast majority of cases of blindness (80.0%), severe visual impairment (67.9%), and visual impairment (87.2%) were avoidable (i.e., due to cataract and its complications, refractive error, trachoma, or corneal scar).

**Table 3:**
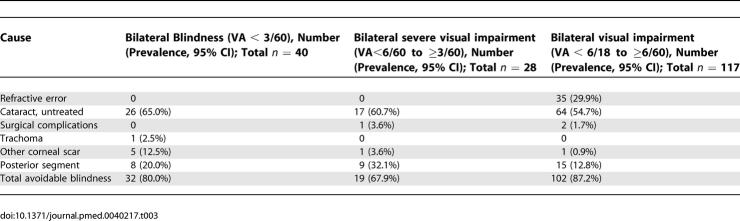
Cause of Blindness, Severe Visual Impairment, and Visual Impairment in People with Available Correction

The cataract surgical coverage was moderate. Approximately four out of every ten people requiring cataract surgery at VA < 6/60 had been operated on, but this proportion fell to one in five people at the VA < 6/18 level (Table 4). The CSC was consistently higher for men than for women at all threshold levels of VA. Lack of awareness of the availability of treatment was the most important barrier to surgery among those who had not undergone surgery (52%), followed by a perceived lack of services (16%), inability to afford the surgery (16%), and lack of a companion (8%).

**Table 4:**
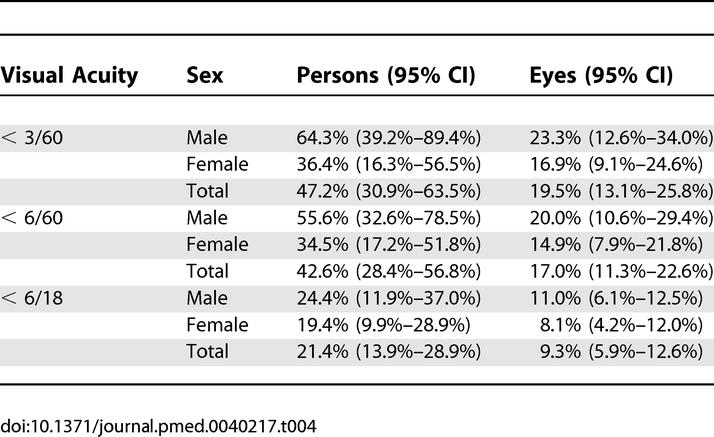
Cataract Surgical Coverage by Person and Eyes in People Aged 50 Years and Older (Best Correction)

Outcome after surgery was poor. Of the 29 eyes that had been operated for cataract, 41% had a poor outcome (VA < 6/60), 35% had a moderate outcome (6/60 ≥ VA < 6/18), and only 24% had a good outcome (VA ≥ 6/18) with available correction (Table 5). The eyes that had received an intraocular lens (IOL) had a better outcome than the eyes that had not. The cause of poor outcomes could be attributable to the surgery in six out of the 12 eyes with a poor outcome, concomitant disease (selection) for three eyes, and sequelae (i.e., posterior capsule opacification) for three eyes. Lack of spectacles was responsible for seven out of the ten eyes with a borderline outcome. Despite the relatively poor outcome, satisfaction with surgery was high: 41% of people were very satisfied with surgery and 28% were partially satisfied, while few people were indifferent (7%), partially dissatisfied (17%), or very dissatisfied (7%).

**Table 5:**
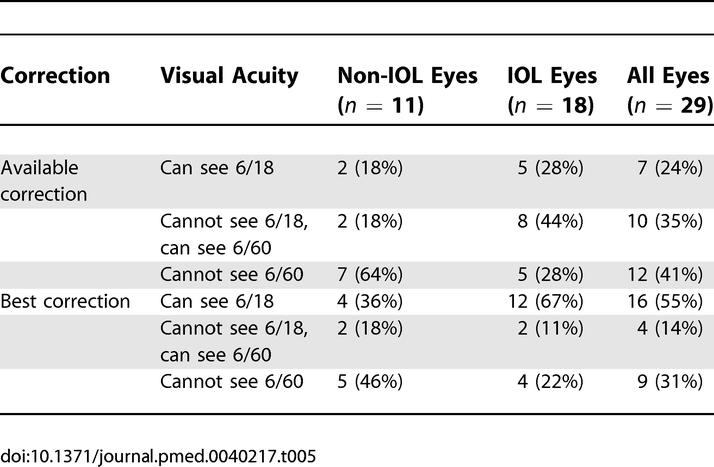
Postoperative Visual Acuity in Eyes Following Cataract Surgery, by IOL Status

### Population Estimates

Extrapolating these estimates to the Western Province population, among the people aged ≥ 50 y there are an estimated 2,565 blind people (95% CI 1,601–3,530) to give an age- and sex-adjusted prevalence of blindness of 1.6% (95% CI 1.0%–2.2%), 1,824 (95% CI 1,095–2,566) severely visually impaired people (1.1%, 95% CI 0.7%–1.6%), and 8,055 (95% CI 6,243–9,870) visually impaired people (4.9%, 95% CI 3.8%–6.0%) (Table 6). There are approximately 1,286 (95% CI 686–1,896) people blind from cataract, 539 (95% CI 245–833) severely visually impaired from cataract, and 4,009 (2,942–5,066) visually impaired from cataract. Using the VISION 2020 target of operating on 20% of the prevalence of cataract per year, and assuming that all people with VA < 6/60 due to cataract require surgery in one eye, then the desired number of cataract surgeries for the Western Province would be at least 365 per year, to give a required cataract surgical rate of minimum 183 or 365 if both eyes were operated.

**Table 6:**
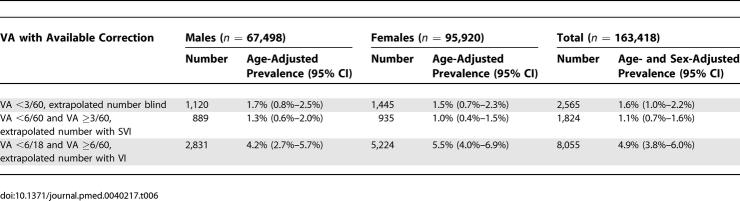
Distribution by Visual Acuity with Available Correction in the Better Eye in Adults Aged 50 Years and Older

On the basis of WHO estimates, we expect that 0.124% of people below the age of 15 y in the Western Province are blind and 0.2% of people aged 15–49 y [1], and the survey results estimate that 1.6% of people aged ≥ 50 y are blind. Assuming that in the Western Province there are approximately 833,000 people aged < 15 y, 984,000 aged 15–49 y, and 163,000 aged ≥ 50 y [7], then across all ages approximately 5,570 are expected to be blind. This gives an overall prevalence of blindness of 0.3% for the population. Alternatively, assuming that 87% of blind people are aged 50 years and older [1], then we would expect there to be 383 people who are blind below the age of 50 years, to give an overall prevalence of blindness of 0.2% for the population.

## Discussion

This survey of blindness in the Western Province of Rwanda found a far lower prevalence of blindness than expected based on current estimates for Africa [1]. This may indicate that blindness is a less pressing public health concern for Rwanda than had previously been assumed. Despite the low prevalence, most of the cases of blindness and visual impairment were avoidable, suggesting that the prevalence can be reduced still further. The relatively low prevalence of cataract blindness may be partly attributable to the moderate cataract surgical coverage. The poor outcome after cataract surgery is of concern. Implementing a monitoring system for cataract surgical results could sensitise surgeons to quality control and help improve outcomes after surgery [11–14]. The provision of spectacles after surgery, improved follow-up after surgery and better selection of patients for surgery will also improve outcomes. The proportion of blindness and visual impairment due to posterior segment disease (including glaucoma and diabetic retinopathy) may grow as avoidable causes are brought under control, and with the continued global epidemic of diabetes, providing an important challenge in the future. The results for the Western Province may be generalisable to most areas in Rwanda (excluding Kigali and the central area) which are characterised by high levels of poverty and poor access to ophthalmic services.

No surveys of blindness had been undertaken in Rwanda before now, but the estimated prevalence from this RAAB was similar to the prevalence of blindness estimated in the national census [7]. The only other RAAB carried out in Africa to date, in Nakuru District, Kenya, also found a lower than expected prevalence of blindness, as only 2.0% of the people aged ≥ 50 y were blind [2]. The causes of blindness were similar, with cataract responsible for the majority of cases followed by posterior segment disease. The only Rapid Assessment of Cataract Surgical Services (RACSS), the precursor to RAAB, in Africa was conducted in a rural area of Cameroon [4]. This survey of people aged ≥ 40 y estimated the prevalence of blindness as only 1.6%—again, the majority of which was caused by cataract. Other surveys conducted in Africa, using a variety of sampling and examination protocols, usually estimated a higher prevalence of blindness, although most found that the main cause is cataract (summarised in a review by Pascolini et al. [15]). Few surveys have been conducted in postconflict areas in Africa. A recent survey from southern Sudan estimated that the prevalence of blindness in the total population was 4.1% [5], although there are some concerns about the methods used in this survey [6]. Another survey conducted in 1998 in a rural area of the Democratic Republic of Congo estimated that only 0.5% of surveyed people over 10 y of age were blind in both eyes [16], is relatively similar to the estimates of blindness obtained for Western Province of Rwanda.

The question remains why the estimated prevalence of blindness was so low in the Western Province of Rwanda. Given that prevalence is a function of incidence and duration of disease, this low prevalence implies that either the incidence of blindness is low or else the duration of blindness is short. The incidence of blindness may be relatively low, since the proportion of the population that is elderly, and therefore at high risk of becoming blind, is small. In Rwanda only 8% of the population is aged ≥ 50 y, compared to 15% in India and 22% in China, and few people were in the oldest age groups. Another reason for the low incidence may be that infectious causes of blindness, which make an important contribution to visual impairment in much of Africa, were virtually absent in this population. There were no cases of blindness from onchocerciasis and only one from trachoma in the sample. It may also be possible that people in Rwanda are less susceptible to developing cataract than people in other regions of the world, although the possible reasons for this are not clear. The duration of blindness may be short, as either people are cured of their blindness or else they have short life expectancy. The CSC, which is a marker of the availability of eye care services, is moderate, suggesting that access to cataract surgical services may contribute to the low prevalence. Mortality rates may have been higher for people who are blind than those in the general population [17], although this is unlikely to be sufficiently marked for people with visual impairment to explain the low prevalence. Western Rwanda was devastated by the genocide in 1994. It is possible that people who were visually impaired or blind were more likely to succumb during the genocide, which could explain part of the low prevalence a decade later, although this explanation is highly speculative. Another possibility is that previous surveys in Africa overestimated the prevalence of blindness, perhaps because they used the random walk method for selecting individuals within the cluster [18], which may have led to an overestimation of the prevalence of blindness if blind people were preferentially included. In contrast, we used compact segment sampling for this RAAB survey [9], which improves objectivity in household selection [19] and avoids other biases inherent in the random walk methodology [20]. Further RAAB surveys are required in Africa, to observe whether the low prevalence of blindness is a consistent finding, and to model the relationship between the prevalence of blindness with age structure and CSC.

The study had many strengths that lend confidence to the estimates obtained. The sampling of the population for the survey was facilitated by a relatively recent census [7]. The Rwandese population is divided into cells for administrative purposes, which approximately corresponded to our desired cluster size. This meant that the sampling was relatively objective, as the village guides were able to inform the team exactly who was eligible within the chosen cluster. There is unlikely to have been serious selection bias, as the response rate was high and the sample was representative of the Western Province population. Information bias in this RAAB was also unlikely, as the ophthalmic examinations were undertaken by experienced ophthalmologists, with acceptable interobserver reliability.

There were also some limitations to the study. The RAAB methodology focuses on the prevalence and causes of visual impairment in people aged ≥ 50 y and so does not produce estimates for childhood blindness or blindness in adults aged less than 50 y. Although only 8% of the population of Rwanda is aged ≥ 50 y, approximately 87% of the cases of blindness in Rwanda are expected to be among people in this age group, and analyses from The Gambia indicate that the causes of blindness and visual impairment in people aged ≥ 50 y gives an accurate estimate of the causes in the total population [21]. Estimation of the magnitude of blindness in the overall population relied on the use of WHO estimates for the prevalence of blindness among those aged < 50 y or else on the assumption of the proportion of blindness in the population aged ≥ 50 y, so the extrapolated results may have been imprecise. Sampling individuals through compact segment sampling is likely to be less subjective than through the random-walk method. However, since not all eligible people in the selected segments were examined (the cluster size was limited to 50 individuals) an element of subjectivity still remained, which may have resulted in bias if it led to the systematic over- or undersampling of blind people. This problem may be overcome in future surveys through the delineation of smaller segments (e.g. five to ten households), with random selection of the number of segments required to make up the estimated sample size per cluster, and inclusion of all eligible persons in all selected segments. If the required sample is not reached, additional segments can be randomly selected (as required) and all eligible persons included. Given that the survey was conducted by visiting households door-to-door in villages, it was not possible to undertake sufficiently detailed ophthalmic examinations to be able to measure posterior segment disorders accurately. The presence of night blindness was not routinely measured in the survey, although anecdotal findings from the ophthalmologists suggest that it was relatively common in this population, so a further study to estimate the prevalence of xerophthalmia may be required. The sample size was relatively small because of the unexpectedly low prevalence of blindness, although this affected only the width of the confidence intervals around the estimates rather than the prevalence estimates. People who had undergone cataract surgery may have been hesitant to express lack of satisfaction with the surgery to health professionals, so satisfaction may have been overestimated.

In conclusion, while the prevalence of blindness in the Western Province of Rwanda was relatively low, most of the cases were avoidable. The implementation of an effective eye care service is a priority and could reduce the prevalence of blindness substantially.

## Abbreviations

CI: confidence interval
CSC: cataract surgical coverage
DEFF: design effect
IOL: intraocular lens
VA: visual acuity
